# Addition of PD-1/PD-L1 inhibitors to chemotherapy for triple-negative breast cancer: a meta-analysis

**DOI:** 10.3389/fonc.2024.1309677

**Published:** 2024-02-09

**Authors:** Juan Yang, Chen Liu, Yaru Guo, Wenwen Guo, Xiaojin Wu

**Affiliations:** Department of Radiation Oncology, The Affiliated Xuzhou Municipal Hospital of Xuzhou Medical University, Xuzhou, Jiangsu, China

**Keywords:** triple-negative breast cancer, PD-1/PD-L1, immune checkpoint inhibitors, chemotherapy, systematic review, meta-analysis

## Abstract

**Background:**

In recent years, the addition of immune checkpoint inhibitors (ICI) to chemotherapy (CT) has become a research hotspot in the therapy of metastatic triple-negative breast cancer. Nevertheless, controversial results have been revealed among the published randomized controlled trials. Hence, a meta-analysis was performed to assess the therapeutic effect of this treatment regimen.

**Methods:**

Five English databases (PubMed, WOS, CENTRAL, Scopus, and Embase), and four Chinese databases (CBM, CNKI, VIP, and Wanfang), as well as oncological meetings, were systematically searched to identify eligible studies that assessed the addition of ICI to CT versus CT alone in metastatic triple-negative breast cancer. The pooled hazard ratios (HR) of progression-free survival (PFS) and overall survival (OS) were estimated using fixed- or random-effect model. Subgroup analyses were also performed in the intention-to-treat (ITT) and PD-L1-positive individuals.

**Results:**

All told there are five eligible randomized controlled trials involving 3,000 patients were enrolled in this meta-analysis. Compared with CT alone, the ICI plus CT regimen significantly increased PFS in the ITT (HR = 0.80, 95% CI: 0.73–0.88) and PD-L1-positive (HR = 0.70, 95% CI: 0.62–0.79) populations, as well as OS in the ITT (HR = 0.89, 95% CI: 0.81–0.97) and PD-L1-positive populations (HR = 0.80, 95% CI: 0.71–0.91). Moreover, the PFS of sufferers treated with the combination strategy of ICI with CT increased alongside PD-L1 enrichment. A clinical benefit in terms of objective response rate was also distinctly observed in both populations treated with ICI plus CT. In the subgroup analysis, patients in the no prior CT subgroup experienced a striking increase in PFS in both populations; however, a difference was not observed in other subgroups.

**Conclusions:**

The combination strategy striking improves PFS and OS in both ITT and PD-L1-positive populations, and PFS is prolonged with PD-L1 enrichment. Patients who do not receive CT prior to this treatment are associated with longer PFS in both populations.

**Systematic review registration:**

https://www.crd.york.ac.uk/PROSPERO/#recordDetails, identifier CRD42021289817.

## 1 Introduction

As reported, the proportion of triple-negative breast cancer (TNBC) in all breast cancer cases was 15%–20% (1). It is a heterogeneous disease associated with more aggressiveness, earlier relapses, and poorer prognosis, in comparison with other types of breast cancer (2, 3). Up till now, there is a lack of therapeutic targets in locally advanced or metastatic TNBC; thus, the therapeutic options for these tumors are limited (1). So far, chemotherapy (CT) is still the only standard systemic therapy in this setting (4), though most patients experience disease progression due to the development of drug resistance (5, 6). Therefore, there is an urgent need for advances in the treatment of TNBC.

Evasion of immune surveillance is the typical hallmark of tumors (7), which suppress immune response primarily via programmed cell death 1 (PD-1) ligand-receptor interaction (8, 9). Over the past decade, immune checkpoint inhibitor (ICI) monotherapy, including PD-1 or programmed cell death-ligand 1 (PD-L1) inhibitor, has revealed hopeful efficacy and tolerable safety by the clinical practice in varieties of solid tumors (10–13). TNBC is characterized by a higher level of tumor-infiltrating lymphocytes (14), more expression of PD-L1 (15), and a larger number of non-synonymous mutations (16), in comparison with other breast cancer subtypes. Considering the stimulatory effect of CT agents on the immune system, the combination treatment strategy of ICI plus CT may be effective against locally advanced or metastatic TNBC (17–19). The IMpassion130 trial was the first clinic study to evaluate the efficacy and tolerance while adding atezolizumab to nanoparticle albumin-bound paclitaxel (nab-paclitaxel) in the standard systemic treatment of locally advanced or metastatic TNBC (20–22). This investigation was followed by other trials, namely IMpassion131 (23), KEYNOTE-355 (24–26), ALICE (27), and TORCHLIGHT (28). While IMpassion130 and KEYNOTE-355 yielded positive results in metastatic TNBC, IMpassion131 failed to certify a distinct clinical benefit. Because of the negative data obtained from IMpassion131, the approval of atezolizumab was also withdrawn by the FDA.

These contrasting findings have caused controversy among researchers. The objective of this meta-analysis was to assess the overall efficacy of ICI plus CT in locally advanced or metastatic TNBC, in comparison with CT alone. To achieve the purpose of providing reliable evidence for the optimization of clinical treatment.

## 2 Methods

This meta-analysis was executed on the basis of the PRISMA 2020 statement (29). The protocol was registered on the international systematic review registry platform of PROSPERO (registration NO: CRD42021289817), as shown in **Supplementary Data 1**.

### 2.1 Literature screening

We systematically searched for eligible literature registered before July 15, 2023 in five English databases (i.e., PubMed, WOS, CENTRAL, Scopus, and Embase databases), and four Chinese databases (i.e., CBM, CNKI, VIP, and Wanfang databases). In addition, oncology annual meetings were also considered. The relevant terms were formulated in accordance with the PICOS principles, including “PD-1”, “PD-L1”, “TNBC”, “immunotherapy”, “chemotherapy”, “ICI”, “Immune Checkpoint Inhibitors”, and corresponding free words. The detailed search strategy can be found in **Supplementary Data 2**.

### 2.2 Inclusion and exclusion criteria

The processes of literature search and selection were independently performed by two researchers (JY and YG), and disputes were resolved by consulting an experienced researcher (WG). The following criteria were utilized for the inclusion of eligible literature (1): patients who were confirmed with locally advanced or metastatic TNBC by histological or cytological examination (2); studies meet the design style of randomized controlled trials (RCTs) (3); the intervention group was composed of ICI and CT, and the control group was CT alone; and (4) availability of complete data on efficacy in progression-free survival (PFS) with hazard ratios (HR), as well as overall survival (OS) outcomes. Exclusion criteria were (1): non-RCTs (e.g., single-arm trials or retrospective studies) (2); patients in the control group were redistributed to the intervention group because of disease progression (3); lack of data on HR with 95% confidence intervals (CI) not only in PFS, but also in OS; and (4) reviews, comments, and case reports.

### 2.3 Information extraction and quality assessment

The related information listed below was extracted by two researchers (JY and YG), individually: trial name, first- or corporate-author, date of publication, trial design, intervention drug of the experimental arm and control arm, sample size, median age, percentage of White/Asian patients, numbers of patient with ECOG performance status (PS) 1/0, percentage of patients with PD-L1 positivity, number of metastatic sites, HR with 95% CI for PFS and OS, odds ratio with 95% CI for objective response rate (ORR), and incidence of all types of adverse events (AEs). Subsequently, cross-checking of the extracted information was performed by two researchers (JY and YG), and controversies were resolved through discussion with a third researcher (WG). Review Manager version 5.4 software was used to assess the risk of bias of enrolled studies. The quality assessment of the enrolled studies depend on the Cochrane Collaboration’s Risk of Bias tool (30). The required information for quality assessment was extracted synchronously with the process of data extraction. The process of quality assessment was also performed by the three aforementioned researchers (independent assessors: JY and YG; arbiter: WG).

### 2.4 Endpoints

The primary efficacy terminations of PFS and OS, were evaluated in intention-to-treat (ITT) population, PD-L1-positive population, and PD-L1-negative population. The secondary efficacy endpoint was ORR, assessed in both above populations. The safety endpoint was the incidence of AEs, including AEs of any grade, AEs of more than 3 grade, immune-related AEs (irAEs), and serious AEs (SAEs). The exploratory subgroup analysis of PFS were carried out based on age, race, ECOG PS, number of metastatic organs, prior neoadjuvant or adjuvant CT, liver metastases, prior taxane treatment, and prior anthracycline treatment. The definition of PFS was the time horizon, which is from the beginning of TNBC patient received treatment to progression of disease according to the RECIST 1.1 or die due to any reason. The definition of OS was the time from the randomization of TNBC patient to death due to any cause. ORR was described as the percentage of patients exhibiting partial or complete remission based on the RECIST 1.1 criteria.

### 2.5 Statistical analysis

The process of data synthesis was conducted by two researchers (JY and WG) using Review Manager 5.4 and STATA 17 software. Wherein, the former was used to merge data of PFS and OS, and the latter was used to evaluate the publication bias of the included studies. The statistical method of generic inverse variance and the pooled effect of HR and 95% CI were used in the estimation of efficacy endpoints. The Mantel–Haenszel statistical method and odds ratio with 95% CI were used to estimate the overall effect on ORR and safety. The heterogeneity among the pooled results was calculated with the chi-squared test and expressed with the value of *I* ^2^ test. The fixed-effect model was used for data analysis when *I* ^2^ > 50%; otherwise, the random-effects model was used. *P* < 0.05 denoted statistical significance for the overall effect.

## 3 Results

### 3.1 Literature screening and research characteristics

**Figure 1:**
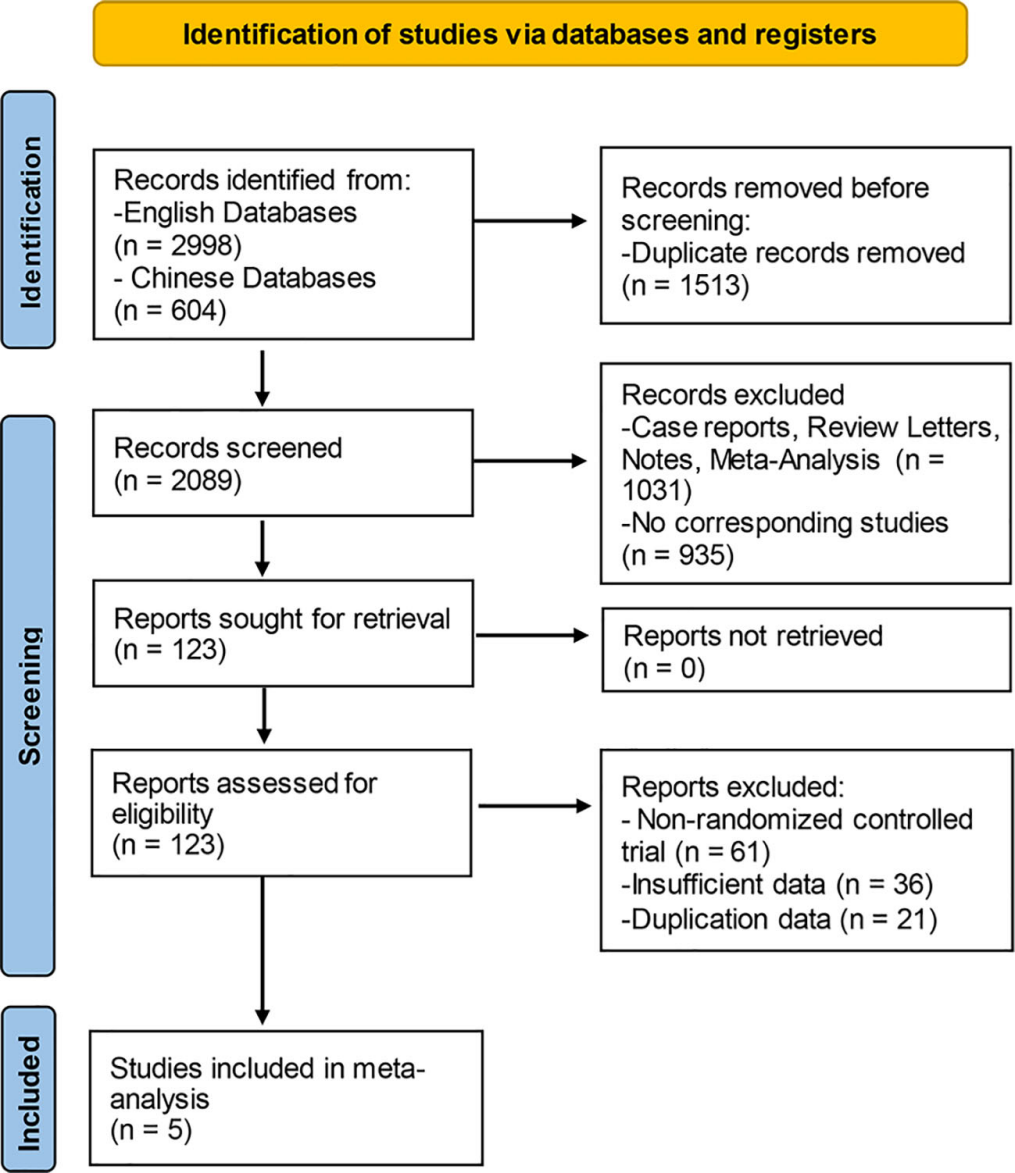
Flowchart of the study selection process for the meta-analysis.

The literature screening was carried out with the Endnote software, and the detailed process was presented in **Figure 1**. In the aggregate, 3,602 documents were identified by means of the systematic literature screening. Among them, 2,998 studies were obtained from English databases; the remaining 604 studies were obtained from the Chinese databases. Prior to screening, 1,501 duplicate records were removed using EndNote (Thomson Corporation, Stanford, USA) software. At the stage of title and abstract screening, totals of 1,914 records were eliminated, including 1,016 records of case reports, review, letters, notes, and meta-analyses, as well as 898 records of non-corresponding studies. At the final stage of full-text reading, 182 reports were excluded in all, including non-RCTs and studies with insufficient or duplicated data. Finally, five eligible studies were enrolled to our meta-analysis altogether.

The essential features of the incorporated trials have been summarized in **Table 1**. Among the five studies, four trials (i.e., IMpassion130, IMpassion131, KEYNOTE-355, and TORCHLIGHT) were double-blind phase III RCTs, while ALICE was a double-blind Phase IIb RCT. There are 3,000 TNBC patients were classified into the experimental arm (1,841 patients who received ICI plus CT) and control arm (1,159 patients who received CT alone). The ICI inhibitors included atezolizumab (anti-PD-L1; used in ALICE, IMpassion130, and IMpassion131), pembrolizumab (anti-PD-1; used in KEYNOTE-355), and toripalimab (anti-PD-1; used in TORCHLIGHT). The CT agents included chemo1 (PEGylated lipoplast adriamycin and low dosage cytoxan, used in ALICE), nab-paclitaxel (used in IMpassion130, KEYNOTE-355, and TORCHLIGHT), paclitaxel (used in IMpassion131 and KEYNOTE-355) or gemcitabine plus carboplatin (used in KEYNOTE-355). The baseline characteristics extracted from the included trials were generally consistent regarding age, race, ECOG PS, PD-L1 positivity, and metastatic sites between the experimental and matched arms.

**Table 1:** Baseline features of the enrolled studies.

Study	Author Year	Trial design Phase Blinding	Intervention drug Experimental arm Control arm	Sample size (n)		Median age (years)	Race White/Asian (%)	ECOG PS 0/1 (%)	PD-L1 positivity (%)	Metastatic sites ≤3 (%)
ALICE	Røssevold 2022 (27)	IIb	Atezolizumab + chemo1	40		58.5	100/0	67.5/32.5	52.5	82.5
		double-blind	Placebo + chemo1	28		52.5	100/0	75.0/25.0	35.7	89.3
IMpassion130	Schmid 2021 (21)	III	Atezolizumab + Nab-P	451		55.0	68.3/18.8	56.9/42.9	41.0	73.8
		double-blind	Placebo + Nab-P	451		56.0	66.7/16.9	60.0/39.8	40.8	75.9
IMpassion131	Miles 2021 (23)	III	Atezolizumab + P	431		54	57/29	61/39	44	76
		double-blind	Placebo + P	221		53	58/30	59/41	46	78
KEYNOTE-355	Cortés 2020 (24)	III	Pembrolizumab + chemo2	566		53	68/22	59/41	75.1	55*
		double-blind	Placebo + chemo2	281		53	69/19	62/38	75.1	59*
TORCHLIGHT	Jiang 2023 (28)	III	Toripalimab + Nab-P	353		53	0/100	48.4/51.6	56.7	88.7
		double-blind	Placebo + Nab-P	178		54.5	0/100	51.1/48.9	56.1	85.4

ECOG PS, ECOG performance status; Nab-P, Nab-paclitaxel; P, paclitaxel; PD-L1, programmed cell death-ligand 1.

chemo1 indicates PEGylated lipoplast adriamycin and low dosage cytoxan; chemo2 indicates Nab-P, paclitaxel, or gemcitabine plus carboplatin.

*Number of metastatic sites: 0–2.

### 3.2 Quality assessment

**Figure 2:**
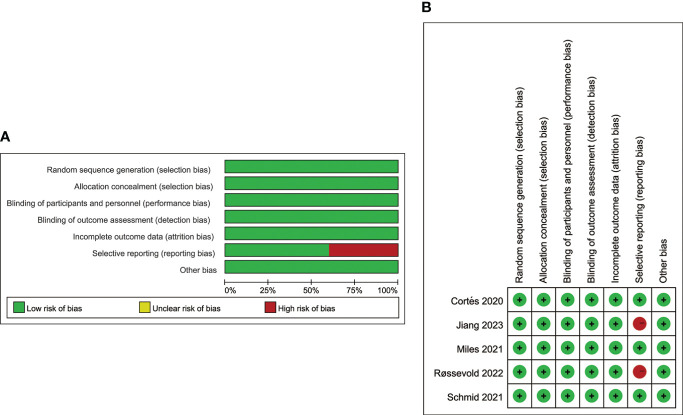
Assessment of the risk of bias. **(A)** Risk of bias graph. **(B)** Risk of bias summary.

As shown in **Figures 2A, B**, the quality assessment results of the enrolled trials are presented with a summary and a detailed graph contained 7 items of evaluation. Overall, all five trials were judged as high-quality investigations. Except for ALICE (Røssevold 2022) and TORCHLIGHT (Jiang 2023), all other trials have low risk of bias. Wherein, the ALICE trial was linked to a high risk of reporting bias because of lacking data on the efficacy of PD-L1-positive and negative subgroups. TORCHLIGHT was linked to a high risk of reporting bias because of insufficient data. It’s worth noting that the data of the TORCHLIGHT trial were extracted from an oral abstract session at the oncology meeting of ASCO (2023).

### 3.3 PFS

#### 3.3.1 Overall evaluation of PFS

**Figure 3:**
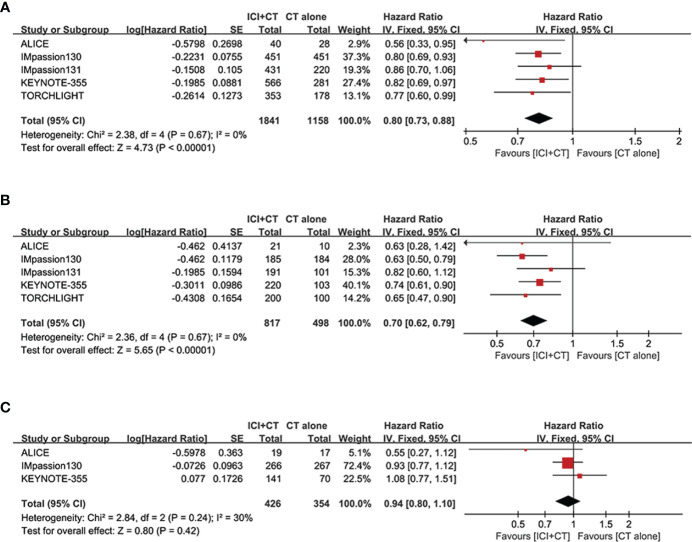
Forest plots of PFS in patients with TNBC treated with ICI plus CT versus CT alone. **(A)** ITT population, **(B)** PD-L1-positive population and **(C)** PD-L1-negative population. CI, confidence interval; CT, chemotherapy; df, degrees of freedom; ICI, immune checkpoint inhibitor; ITT, intention-to-treat; PD-L1, programmed cell death-ligand 1; PFS, progression-free survival; SE, standard error; TNBC, triple-negative breast cancer.

The pooled HR of PFS were estimated in the ITT population (five trials involving 3,000 patients), PD-L1-positive population (five trials involving 1,628 patients) and PD-L1-negative population (three trials involving 780 patients). The overall evaluation in both the ITT and PD-L1-positive populations consistently showed a significant benefit following the treatment of ICI plus CT (ITT: HR = 0.80, 95% CI: 0.73–0.88, *P* < 0.05 (**Figure 3A**); PD-L1-positive: HR = 0.80, 95% CI: 0.73–0.88, *P* < 0.05 (**Figure 3B**), without heterogeneity (*I* ^2^ = 0). Nevertheless, pooled result in the PD-L1 negative individuals did not show striking difference in two groups (HR = 0.94, 95% CI: 0.80–1.10, *P* = 0.42 (**Figure 3C**), with moderate heterogeneity (*I* ^2^ = 30%).

**Figure 4:**
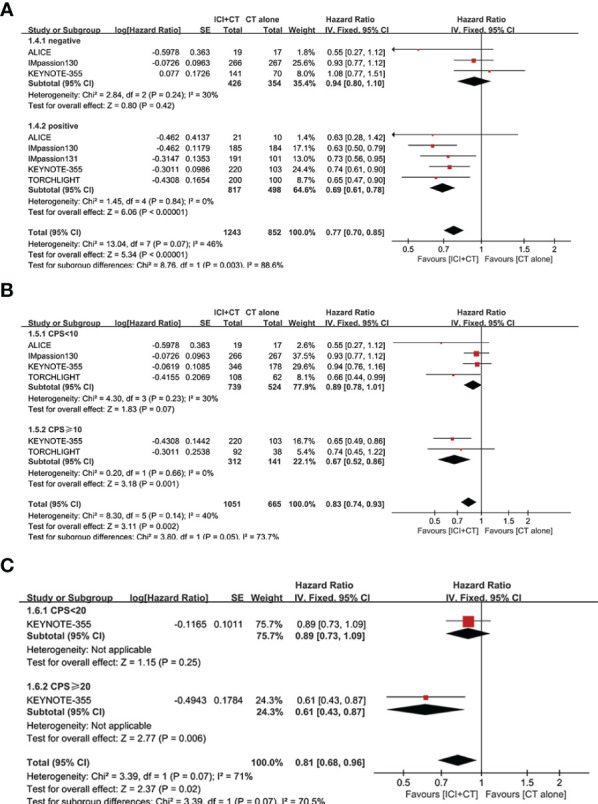
Exploratory analysis of PFS between the magnitude of benefit and PD-L1 enrichment for ICI plus CT versus CT alone. **(A)** PD-L1-negative population *vs*. PD-L1-positive population. **(B)** CPS < 10 *vs*. CPS ≥ 10. **(C)** CPS < 20 *vs*. CPS ≥ 20. CI, confidence interval; CPS, combined positive score; df, degrees of freedom; ICI, immune checkpoint inhibitor; PD-L1, programmed cell death-ligand 1; PFS, progression-free survival; SE, standard error.

We sought to explore the correlation between the magnitude of PFS benefit of this combination regimen and degree of PD-L1 enrichment in TNBC patients. For this purpose, a combined HR evaluation of PFS was conducted according to the PD-L1 enrichment at baseline. As shown in **Figures 4A–C**, ICI plus CT improved PFS with the enrichment of PD-L1 in TNBC patients.

#### 3.3.2 Subgroup analysis of PFS

To compare the curative effect of ICI plus CT in patients with different baseline characteristics, exploratory subgroup analysis was conducted in PD-L1-positive patients, which were based on age, race, ECOG PS, metastatic sites, liver metastases, prior CT, prior taxane treatment, and prior anthracycline treatment.

**Figure 5:**
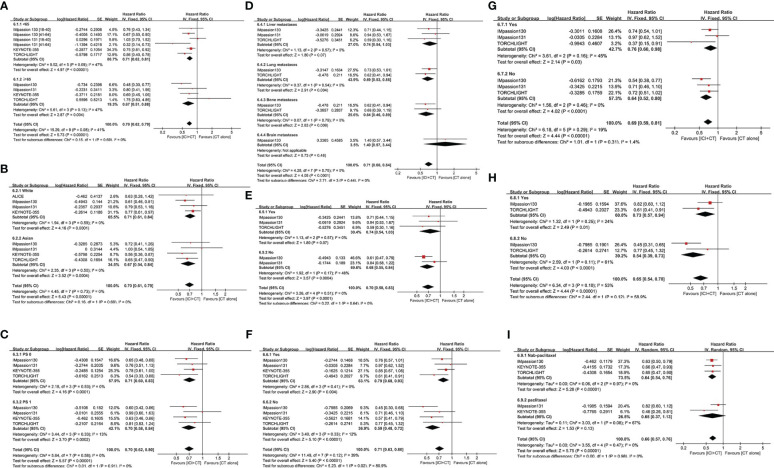
Subgroup analysis of PFS in the PD-L1-positive population. **(A)** Age. **(B)** Race. **(C)** ECOG PS. **(D)** Metastatic sites. **(E)** Presence or absence of liver metastasis. **(F)** Use or no use of prior CT. **(G)** Use or no use of prior taxane treatment. **(H)** Use or no use of prior anthracycline treatment. **(I)** Nab-paclitaxel *vs*. paclitaxel. CI, confidence interval; CT, chemotherapy; df, degrees of freedom; ECOG PS, Eastern Cooperative Oncology Group performance status; nab-paclitaxel, nanoparticle albumin-bound paclitaxel; PD-L1, programmed cell death-ligand 1; PFS, progression-free survival; SE, standard error.

After treatment of ICI plus CT, both of two age subgroups achieved longer PFS (age 18–64 years: HR = 0.71, 95% CI: 0.62–0.81, *I* ^2^ = 47%; age ≥ 65 years: HR = 0.67, 95% CI:0.51–0.88, *I* ^2^ = 47%), showed no significant difference (*P* = 0.69) (**Figure 5A**). Equivalent PFS benefits were achieved in race subgroups (White: HR = 0.71, 95% CI: 0.61–0.84, *I* ^2^ = 0; Asian: HR = 0.67, 95% CI: 0.54–0.84, *I* ^2^ = 0; *P* = 0.69) (**Figure 5B**), and ECOG PS subgroups (PS 0: HR = 0.71, 95% CI: 0.60–0.83, *I* ^2^ = 0; PS 1: HR = 0.70, 95% CI: 0.58–0.84, *I* ^2^ = 13%; *P* = 0.91) (**Figure 5C**).

In the subgroup analysis of PFS with regard to different metastasis sites, patients with lung or bone metastases experienced remarkable improvement in PFS after the addition of ICI to CT (lung metastases: HR = 0.69, 95% CI: 0.53–0.88; bone metastases: HR = 0.64, 95% CI: 0.46–0.89; both without heterogeneity: *I* ^2^ = 0) (**Figure 5D**). On the contrary, those with liver or brain metastases did not achieve a distinct advantage in PFS (liver metastases: HR = 0.74, 95% CI: 0.54–1.03; brain metastases: HR = 1.40, 95% CI: 0.57–3.44) following a combination of ICI and CT compared with CT alone. However, patients without liver metastases were linked to better PFS versus those with liver metastases (**Figure 5E**).

To explore the influence of prior CT regimens on the effect of ICI plus CT, a special subgroup analysis was conducted (**Figure 5F**). Compared with patients who obtained CT alone previously (HR = 0.79, 95% CI: 0.68–0.93, *I* ^2^ = 0), those who did not receive it experienced obvious enhancement in PFS (HR = 0.59, 95% CI: 0.48–0.72, *I* ^2^ = 12%) (*P* = 0.02). The funny fact is that there was no real distinction among PD-L1-positive patients who had ever received or not received taxane treatment (Yes: HR = 0.76, 95% CI: 0.60–0.98; No: HR = 0.64, 95% CI: 0.52–0.80; *P* = 0.31) (**Figure 5G**), as well as among those who had received or not received anthracycline treatment (Yes: HR = 0.73, 95% CI: 0.57–0.94; No: HR = 0.54, 95% CI: 0.39–0.73; *P* = 0.12) (**Figure 5H**). In addition, the subgroup analysis of PFS in patients who had previously received nab-paclitaxel or paclitaxel also did not reveal a significant difference (**Figure 5I**).

### 3.4 OS and ORR

**Figure 6:**
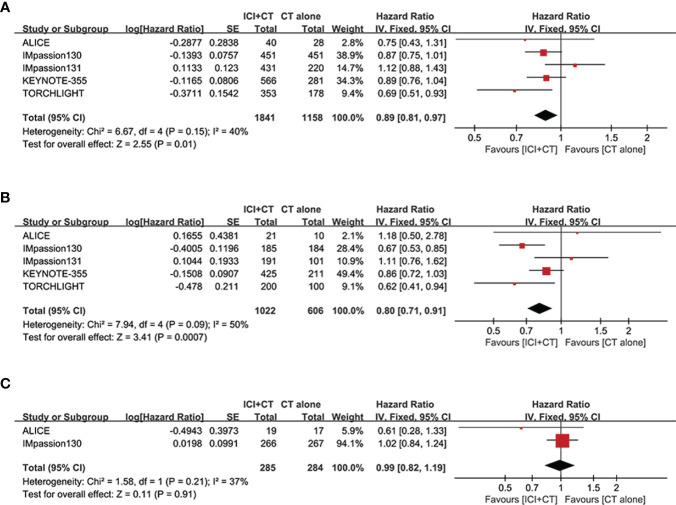
Forest plots of OS in patients with TNBC treated with ICI plus CT versus CT alone. **(A)** ITT population. **(B)** PD-L1-positive population. **(C)** PD-L1-negative population. CI, confidence interval; CT, chemotherapy; df, degrees of freedom; ICI, immune checkpoint inhibitor; ITT, intention-to-treat; OS, overall survival; PD-L1, programmed cell death-ligand 1; SE, standard error; TNBC, triple-negative breast cancer.

**Figure 7:**
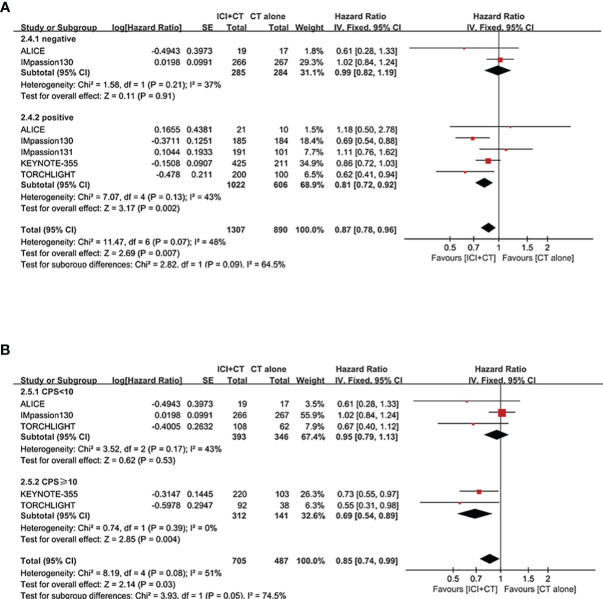
Exploratory analysis of OS between the magnitude of benefit and PD-L1 enrichment for ICI plus CT versus CT alone. **(A)** PD-L1-negative population *vs*. PD-L1-positive population. **(B)** CPS < 10 *vs*. CPS ≥ 10. CI, confidence interval; CPS, combined positive score; CT, chemotherapy; df, degrees of freedom; ICI, immune checkpoint inhibitor; OS, overall survival; PD-L1, programmed cell death-ligand 1; SE, standard error; TNBC, triple-negative breast cancer.

We evaluated OS in the ITT population (five trials involving 3,000 patients), PD-L1-positive population (five trials involving 1,628 patients), and PD-L1-negative population (two trials involving 569 patients). A significant increase of OS was achieved not only in the ITT population (HR = 0.89, 95% CI: 0.81–0.97, *P* < 0.05), with moderate heterogeneity (*I* ^2^ = 40%, as shown in **Figure 6A**), but in the PD-L1-positive population (HR = 0.80, 95% CI: 0.71–0.91, *P* < 0.05, **Figure 6B**), with moderate heterogeneity (*I* ^2^ = 50%). Considering some of the OS data are not yet mature in the TORCHLIGHT study, an exploratory analysis was conducted by removing the TORCHLIGHT study. The overall evaluation in the ITT and PD-L1-positive populations still showed a benefit following the treatment of ICI plus CT (ITT: HR = 0.91, 95% CI: 0.83–1.00, **Supplementary Figure 1A**; PD-L1-positive: HR = 0.83, 95% CI: 0.72–0.94, **Supplementary Figure 1B**). In the PD-L1-negative individuals, however, there was no improvement on OS (HR = 0.99, 95% CI: 0.82–1.19, *P* = 0.91, as shown in **Figure 6C**), with moderate heterogeneity (*I* ^2^ = 37%). Correlation analysis between this combination strategy and the degree of PD-L1 enrichment was also conducted in OS (**Figures 7A, B**). The results indicated an increasing trend in OS with PD-L1 enrichment.

In addition, the secondary efficacy endpoint of ORR was also assessed in both populations. We evaluated ORR in the ITT population (four trials involving 2,468 patients), PD-L1-positive population (three trials involving 1,297 patients). A remarkably increase of ORR was achieved not only in the ITT population (OR = 1.35, 95% CI: 1.15–1.60, *P* < 0.05), without heterogeneity (*I* ^2^ = 0, as shown in **Supplementary Figure 2A**), but in the PD-L1-positive population (OR = 1.48, 95% CI: 1.18–1.86, *P* < 0.05), with mild heterogeneity (*I* ^2^ = 20%, as shown in **Supplementary Figure 2B**).

### 3.5 Safety

**Figure 8:**
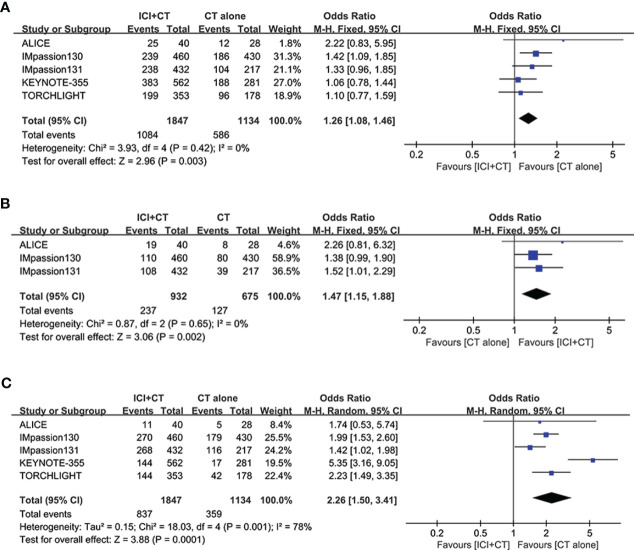
Safety analysis in the ITT population. **(A)** Grade ≥ 3 adverse events. **(B)** Serious adverse events. **(C)** Immune-related adverse events. CI, confidence interval; df, degrees of freedom; ITT, intention-to-treat; SE, standard error.

Five studies involving 2,981 patients (ITT populations) were included in this safety analysis regarding grade ≥ 3 AEs, SAEs, and irAEs. As shown in **Figures 8A**, in comparison with patients treated with CT alone, patients treated with ICI plus CT have significantly higher rate of grade ≥ 3 AEs (grade ≥ 3 AEs: odds ratio = 1.26, 95% CI: 1.08–1.46, *P* < 0.05), without heterogeneity (*I* ^2^ = 0). Similar results of SAEs are shown in **Figure 8B**: odds ratio = 1.47, 95% CI: 1.15–1.88, *P* < 0.05. In addition, a significantly higher incidence of irAEs was also showed in patients who had received ICI plus CT versus those who received CT alone (odds ratio = 2.26, 95% CI: 1.50–3.41, *P* < 0.05), though followed with high heterogeneity (*I* ^2 ^= 78%, **Figure 8C**).

### 3.6 Publication bias

**Figure 9:**
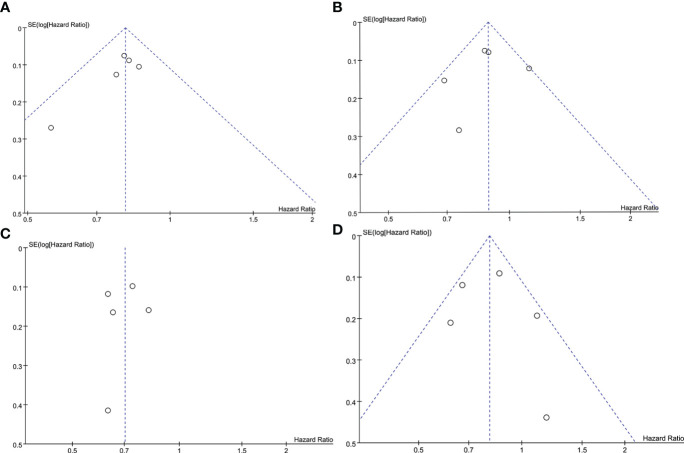
Funnel plot of the PFS and OS analyses. **(A)** PFS in the ITT population. **(B)** PFS in the PD-L1-positive population. **(C)** OS in the ITT population. **(D)** OS in the PD-L1-positive population. ITT, intention-to-treat; OS, overall survival; PD-L1, programmed cell death-ligand 1; PFS, progression-free survival; SE, standard error.

To judge the presence or absence of publication bias in PFS and OS, funnel plots of PFS and OS were respectively presented in **Figures 9A–C** using Review Manager 5.4. As shown in **Figure 9A**, the funnel plot of PFS in the ITT population is visually symmetrical, as well as the PD-L1-positive individuals (**Figure 9B**). The funnel plots of OS in both populations are presented in **Figures 9C, D**, which also make the similar conclusion. Additionally, to identify the publication bias numerically, Egger’s test was carried out through STATA 17 software, with all the *P* values more than 0.05.

## 4 Discussion

As is well-known, CT brought limited effectiveness against locally advanced or metastatic TNBC, though it was recommended as first-line standard systemic treatment. Due to the inevitable drug-resistance of CT, there is a crying need for advances in the treatment regimen of TNBC patients Recently, a series of controversial results have been published, and there is no consensus on the usefulness of the combination regimen of ICI plus CT for patients with TNBC. Hence, there is a need to clarify the concrete effects of the addition of ICI to CT in this setting.

This is the most comprehensive meta-analysis conducted thus far, evaluating the efficacy and safety of combining ICI with CT as standard systemic therapeutic regimen for locally advanced or metastatic TNBC. The meta-analysis confirmed that the combination of ICI and CT obviously enhanced PFS in both the ITT and PD-L1-positive populations, in comparison with CT alone. Nevertheless, there was no difference observed between PD-L1-negative patient populations. Furthermore, the magnitude of PFS benefit increased with PD-L1 enrichment. More critically, significantly improved OS was also observed in both populations; In the PD-L1-negative population, however, there was no substantial difference was noted. The subgroup analysis of PFS revealed that patients with certain metastatic sites (i.e., lung and bone metastases) or who had previously received CT regimens experienced a significant improvement in PFS. On the contrary, there were no differences observed based on the baseline characteristics with respect to race, age, ECOG PS, metastatic sites (liver and brain metastases), and prior taxane or anthracycline treatment.

Similar with PFS, as the enrichment of PD-L1, so will the magnitude of OS benefit. The exploratory results revealed a clear trend toward improved outcomes (including PFS and OS) with PD-L1 enrichment. This finding is consistent with those of earlier trials of pembrolizumab monotherapy. In the KEYNOTE-086 trial which was focusing on pembrolizumab monotherapy, a tendency towards an increased response rate was observed among PD-L1-positive population, in comparison with those with PD-L1-negative TNBC (10, 11). A clear tendency towards increased treatment effect was also observed with PD-L1 enrichment in the KEYNOTE-119 trial (12), especially in PD-L1-positive population with a combined positive score ≥ 20. Collectively, these findings emphasized the necessity of PD-L1 status detection in patients with TNBC.

Considering the negative outcomes obtained from the IMpassion131 trial, the FDA withdrew its authorization for the combination of atezolizumab with paclitaxel in August 2021 (23). In contrast, the positive outcomes from the IMpassion130 and ALICE studies showed a significantly improved treatment effect on patients with TNBC following the addition of atezolizumab to CT versus CT alone (21, 22, 27). Distinct from paclitaxel used as systemic CT regimens in the IMpassion131 trial, albumin bound-paclitaxel was utilized in the IMpassion130 trial. And PEGylated lipoplast Adriamycin combined with low dosage cytoxan were used in the ALICE trials. In PD-L1-positive population, quite interestingly, the subgroup analysis for PFS did not reveal a difference between nab-paclitaxel and paclitaxel (nab-paclitaxel: HR = 0.64, 95% CI: 0.54–0.76; paclitaxel: HR = 0.65, 95% CI: 0.37–1.13; *P* = 0.98) (**Figure 5I**), despite the high heterogeneity detected across the trials using paclitaxel. To our knowledge, CT agents could promote the release of cancer cell antigens by killing cancer cells (31).

Recently, several meta-analyses have been carried out to assess the overall efficacy of ICI plus CT. Villacampa G et al. concluded that ICI plus CT significantly improved PFS in ITT and PD-L1-positive populations, while no significant OS benefit was observed in ITT population, though a tendency towards improved OS in PD-L1-positive population (32). The meta-analysis from Zhang W et al. indicated that ICI combined with CT can improve PFS in both populations, but have no efficacy on OS in either population (33). Similarly, a tendency toward enhanced PFS and OS was observed in ITT population by Liang X et al, but without significant differences (34). What is noteworthy is that the numbers of enrolled study of meta-analysis from Villacampa G et al. is 3, conclusion from it needed to be further verified in larger trials and patients. And the intervention drug of experimental arm of meta-analysis from Zhang W et al. include not only combination strategy of ICI plus CT, but ICI monotherapy. Different from the above studies, our study drew a conclusion that the combination strategy striking improves PFS and OS in both ITT and PD-L1-positive populations, based on more RCTs, more consistent baseline characteristics of patient and strictly limited intervention measure. This meta-analysis demonstrated that the combination of ICI with CT achieved 20% and 30% relative decrease in the incidence of progressive disease in the ITT and PD-L1-positive populations, respectively. More importantly, 11% and 20% relative reduction in the risk of death was also achieved in those populations, respectively. These data demonstrate the clinical validity of ICI combined with CT as a standard treatment regimen in PD-L1-positive patients with locally advanced or metastatic TNBC, in comparison with CT alone.

The subgroup analysis of PFS according to baseline characteristics of patients presented equivalent PFS benefit. These findings indicated that the baseline characteristics (i.e., age < 65 or ≥ 65 years, White or Asian race, and ECOG PS of 0 or 1) did not exert a significant effect. Interestingly, patients who have not previously received (neo)adjuvant CT gained a longer PFS benefit compared with those who had received prior (neo)adjuvant CT. Despite the absence of obvious statistics difference between individuals who had previously received or not received taxane/anthracycline treatment, a longer PFS benefit was observed in the latter group. These results might be explained by the higher immunogenicity detected in untreated patients with TNBC (10, 11, 35), to a certain extent.

The safety profile of combining ICI with CT was generally consistent with the available safety data for the individual agents, without evidence of new safety signals and cumulative toxicity. The intervention group of ICI plus CT experienced higher incidence of grade ≥ 3 AEs (HR = 1.26, 95% CI: 1.08–1.46, *P* < 0.05), in comparison with the control group. Similar differences were observed in the comparison of SAEs of any grade (HR = 1.47, 95% CI: 1.15–1.88, *P* < 0.05), and in the comparison of irAEs of any grade (HR = 2.26, 95% CI: 1.50–3.41, *P* < 0.05). These findings may be explained by the toxic effects of ICI and ICI–CT interactions.

It is inevitable that there are some restrictions under this meta-analysis. Firstly, different kinds of ICI (e.g., atezolizumab, pembrolizumab, and toripalimab) were used in the five trials included in this analysis, as well as all kinds of CT regimens. Secondly, the detection method of PD-L1 status are different between KEYNOTE 355 trial (IHC 22C3 pharmDx assay, combined positive score ≥ 1) and the other trials (VENTANA PD-L1 (SP142) Assay, immune cells ≥ 1%). Thirdly, restricted by the small sample size in the ALICE trial and insufficient subgroup data on OS in ALICE, IMpassion131, and TORCHLIGHT trials, researchers ought to be more cautious to interpret these findings.

In summary, this meta-analysis concluded that the combination of ICI with CT striking enhanced the PFS and OS in patients with locally advanced or metastatic TNBC. These clinical benefits achieved in both the ITT and PD-L1-positive individuals, regardless of age, race, or ECOG PS. A superior efficacy was achieved in patients that had never received (neo)adjuvant CT previously, and we also a trend towards longer PFS was realized in patients that had never received taxane/anthracycline treatment. However, the combination regimen of combining ICI with nab-paclitaxel or paclitaxel exerted equivalent effect. Considering the encouraging effectiveness and acceptable safety profile, the combination of ICI plus CT may be recommended for use in clinical practice.

## Data availability statement

The original contributions presented in the study are included in the article/**Supplementary Material**. Further inquiries can be directed to the corresponding author.

## Author contributions

JY: Data curation, Formal analysis, Investigation, Methodology, Resources, Software, Writing – original draft, Writing – review & editing. CL: Methodology, Resources, Software, Supervision, Writing – review & editing. YG: Data curation, Methodology, Resources, Software, Writing – review & editing. WG: Methodology, Resources, Software, Visualization, Writing – review & editing. XW: Conceptualization, Funding acquisition, Methodology, Supervision, Validation, Writing – review & editing.
