# Supplementary material for: Addition of PD-1/PD-L1 inhibitors to chemotherapy for triple-negative breast cancer: a meta-analysis
Source: Front Oncol. 2024 Feb 9;14:1309677. doi: 10.3389/fonc.2024.1309677 (PMC10884307; doi:10.3389/fonc.2024.1309677)
Supplement: Supplementary file 1 [file DataSheet_1.pdf]

Systematic review

Please select one of the options below to edit your record. Either option will create a new version of the record - the existing version will remain unchanged.

A list of fields that can be edited in an update can be found here

1. * Review title. [1 change]

Give the title of the review in English

PD-1/PD-L1 immune checkpoint inhibitors addition to chemotherapy in locally advanced or metastatic triple-negative breast cancer: A systematic review and meta-analysis

2. Original language title. [1 change]

For reviews in languages other than English, give the title in the original language. This will be displayed with the English language title.

免疫治疗联合化疗相比安慰剂联合化疗应用于局部晚期或转移性三阴性乳腺癌的系统评价和荟萃分析

3. * Anticipated or actual start date.

Give the date the systematic review started or is expected to start.

10/10/2021

4. * Anticipated completion date. [2 changes]

Give the date by which the review is expected to be completed.

10/10/2023

5. * Stage of review at time of this submission. [2 changes]

This field uses answers to initial screening questions. It cannot be edited until after registration.

Tick the boxes to show which review tasks have been started and which have been completed.

Update this field each time any amendments are made to a published record.

The review has not yet started: No

Review stage	Started	Completed
Preliminary searches	Yes	Yes
Piloting of the study selection process	Yes	Yes
Formal screening of search results against eligibility criteria	Yes	Yes
Data extraction	Yes	Yes
Risk of bias (quality) assessment	No	No
Data analysis	No	No

Provide any other relevant information about the stage of the review here.

6. * Named contact. [1 change]

The named contact is the guarantor for the accuracy of the information in the register record. This may be any member of the review team.

Juan Yang

Email salutation (e.g. "Dr Smith" or "Joanne") for correspondence:
Miss Yang

7. * Named contact email. [1 change]

Give the electronic email address of the named contact.

982320565@qq.com

8. Named contact address

PLEASE NOTE this information will be published in the PROSPERO record so please do not enter private information, i.e. personal home address [1 change]

Give the full institutional/organisational postal address for the named contact.

Department of Radiation Oncology, Affiliated Municipal Hospital of Xuzhou Medical University, Xuzhou 221000, China.

9. Named contact phone number. [1 change]

Give the telephone number for the named contact, including international dialling code.

+8618936651096

10. * Organisational affiliation of the review. [1 change]

Full title of the organisational affiliations for this review and website address if available. This field may be completed as 'None' if the review is not affiliated to any organisation.

Department of Radiation Oncology, Affiliated Municipal Hospital of Xuzhou Medical University

Organisation web address:
https://www.xzhmu.edu.cn/

11. * Review team members and their organisational affiliations. [1 change]

Give the personal details and the organisational affiliations of each member of the review team. Affiliation refers to groups or organisations to which review team members belong.
NOTE: email and country now MUST be entered for each person, unless you are amending a published record.

- Miss Juan Yang. Department of Radiation Oncology, Affiliated Municipal Hospital of Xuzhou Medical University
- Miss Yaru Guo. Department of Radiation Oncology, Affiliated Municipal Hospital of Xuzhou Medical University
- Mrs Wenwen Guo. Department of Radiation Oncology, Affiliated Municipal Hospital of Xuzhou Medical University
- Professor Xiaojin Wu. Department of Radiation Oncology, Affiliated Municipal Hospital of Xuzhou Medical University

12. * Funding sources/sponsors.

Details of the individuals, organizations, groups, companies or other legal entities who have funded or sponsored the review.

No funding was provided for this work

Grant number(s)
State the funder, grant or award number and the date of award

13. * Conflicts of interest.

List actual or perceived conflicts of interest (financial or academic).

None

The authors declare no conflict of interest.

14. Collaborators.

Give the name and affiliation of any individuals or organisations who are working on the review but who are not listed as review team members. **NOTE: email and country must be completed for each person, unless you are amending a published record.**

15. * Review question. [1 change]

State the review question(s) clearly and precisely. It may be appropriate to break very broad questions down into a series of related more specific questions. Questions may be framed or refined using PI(E)COS or similar where relevant.

The Efficacy and Safety of immunotherapy plus chemotherapy for patients with locally advanced or metastatic triple-negative breast cancer compared to placebo plus chemotherapy

16. * Searches. [1 change]

State the sources that will be searched (e.g. Medline). Give the search dates, and any restrictions (e.g. language or publication date). Do NOT enter the full search strategy (it may be provided as a link or attachment below.)

Sources that will be used to identify studies for the systematic review: PubMed, Embase, CENTRAL, Web of Science, CNKI, CBM, Wanfang and VIP databases, as well as oncological meetings.
Search dates: from the inception of each database to June 14, 2023.
Restrictions on the search language: English.
Searches will be re-run prior to the final analysis.
Exclude ongoing studies with results not presented nor published at the time of the literature search.

17. URL to search strategy. [1 change]

Upload a file with your search strategy, or an example of a search strategy for a specific database, (including the keywords) in pdf or word format. In doing so you are consenting to the file being made publicly accessible.

Or provide a URL or link to the strategy. Do NOT provide links to your search **results**.

https://www.crd.york.ac.uk/PROSPEROFILES/289817_STRATEGY_20211106.pdf

Do not make this file publicly available until the review is complete

18. * Condition or domain being studied. [1 change]

Give a short description of the disease, condition or healthcare domain being studied in your systematic review.

Locally advanced or metastatic triple-negative breast cancer

19. * Participants/population.

Specify the participants or populations being studied in the review. The preferred format includes details of both inclusion and exclusion criteria.

Inclusion: Patients were aged 18 years or older and had histologically documented, unresectable, locally advanced or metastatic triple-negative breast cancer.
Exclusion: Known symptomatic CNS disease, prior immunotherapy and history of autoimmune disease.

20. * Intervention(s), exposure(s).

Give full and clear descriptions or definitions of the interventions or the exposures to be reviewed. The preferred format includes details of both inclusion and exclusion criteria.

The experimental arm interventions are defined as combining programmed cell death-1 (PD-1) or programmed cell death-ligand1 PD-L1(PD-L1) blockade with the chosen first-line chemotherapy for unresectable, locally advanced or metastatic triple-negative breast cancer until progression, unacceptable toxicity or withdrawal.
Examples include combining atezolizumab with first-line nab-paclitaxel, combining atezolizumab with first-line chemotherapy (capecitabine or gemcitabine/carboplatin), first-line pembrolizumab (pembro) + chemotherapy (chemo) and so on.

21. * Comparator(s)/control.

Where relevant, give details of the alternatives against which the intervention/exposure will be compared (e.g. another intervention or a non-exposed control group). The preferred format includes details of both inclusion and exclusion criteria.

Patients received chemotherapy alone in the control arm until progression or unacceptable toxicity.
The control arm interventions are defined as combining placebo with the chosen first-line chemotherapy or first-line chemotherapy alone for unresectable, locally advanced or metastatic triple-negative breast cancer until progression, unacceptable toxicity or withdrawal.
Examples include combining placebo with first-line nab-paclitaxel, first-line chemotherapy (capecitabine or gemcitabine/carboplatin) alone, placebo + chemotherapy (chemo) and so on.

22. * Types of study to be included. [1 change]

Give details of the study designs (e.g. RCT) that are eligible for inclusion in the review. The preferred format includes both inclusion and exclusion criteria. If there are no restrictions on the types of study, this should be stated.

Articles should be randomized, placebo-controlled studies in patients with advanced triple negative breast cancer.

23. Context.

Give summary details of the setting or other relevant characteristics, which help define the inclusion or exclusion criteria.

24. * Main outcome(s). [1 change]

Give the pre-specified main (most important) outcomes of the review, including details of how the outcome is defined and measured and when these measurement are made, if these are part of the review inclusion criteria.

Progression-free survival (PFS, defined as the time from randomization to the time of radiographic progression (as assessed by irRECIST) or death from any cause during the study Timepoint(s) of evaluation of this end point, measured using Response Evaluation Criteria in Solid Tumors [RECIST] v1.1. Overall survival (OS, defined as the time from the date of randomization to the date of death from any cause)

Measures of effect
hazard ratios

25. * Additional outcome(s). [1 change]

List the pre-specified additional outcomes of the review, with a similar level of detail to that required for main outcomes. Where there are no additional outcomes please state 'None' or 'Not applicable' as appropriate to the review

Overall response rate (ORR, defined as the proportion of patients with an objective tumor response (either partial response [PR] or complete response [CR] per investigator using irRECIST))

Measures of effect
Odds ratios

26. * Data extraction (selection and coding). [1 change]

Describe how studies will be selected for inclusion. State what data will be extracted or obtained. State how this will be done and recorded.

Study selection

Two investigators (Yang, Guo) will independently double-screen and review the list of records retrieved in accordance, to identify potentially eligible articles.
When discrepant opinions on study selection among investigators occurred, a third author (Wu) will functioned as tiebreaker; when no reconciliation still reached, all the authors will be consulted.
Data extraction
Data extracted include: study name, first author, year of publication, study design, study phase, number of patients enrolled, number of TNBC patients enrolled, PD-L1 status, anti-PD1 or anti-PD-L1 agent studied, regimen of CT, rate of patients achieving PFS, OS, ORR in experimental and control arms, according to PD-L1 status and according to nodal status and/or disease stage. Toxicity profile in experimental and control arms were also extracted, in terms of grade 3 or 4 adverse events (AEs) rate, serious adverse events (SAE) rate, immune-related AEs and treatment discontinuation rate.
Yang and Guo independently extracted data from the studies and all discrepancies were resolved by consensus with all investigators.
When duplicate publications of trials were identified, only the most recent and complete report was included.
The missing data will be handled through contacting with study investigators for unreported data or additional details.
The data is recorded in an excel spreadsheet.
The software or tool, that will be used for data extraction and management, are https://apps.automeris.io /wpd/index.zh_CN.html and Review Manager.

27. * Risk of bias (quality) assessment.

State which characteristics of the studies will be assessed and/or any formal risk of bias/quality assessment tools that will be used.

The Cochrane risk of bias tool, which is Review Manager, will be used to assess the characteristics of the included studies, include random sequence generation (selection bias), allocation concealment (selection bias), blinding of participants and personnel (performance bias), blinding of outcome assessment (detection bias), incomplete outcome data (attrition bias), selective reporting (reporting bias) and other bias.
The assessment will be done at outcome level.
Two reviewers will be involved in the assessment of the Risk of bias of the included studies and all disagreements will be resolved by consensus with all investigators.

28. * Strategy for data synthesis. [1 change]

Describe the methods you plan to use to synthesise data. This **must not be generic text** but should be **specific to your review** and describe how the proposed approach will be applied to your data.
If meta-analysis is planned, describe the models to be used, methods to explore statistical heterogeneity, and software package to be used.

The minimum number of studies is two trials required for synthesis.
Data will be synthesised including hazard ratios (HR) and corresponding 95 % confidence intervals (CI) for the association between treatment (adjuvant chemotherapy with or without immunotherapy) and PFS, OS as well as toxicity.
HR for individual studies will be combined using a fixed- or random-effects meta-analysis.

29. * Analysis of subgroups or subsets. [1 change]

State any planned investigation of 'subgroups'. Be clear and specific about which type of study or participant will be included in each group or covariate investigated. State the planned analytic approach.

Immune checkpoint inhibitor (ICI) blocks the binding of PD-L1 to its receptors PD-1 and B7.1, thus restoring tumor-specific T cell immunity. TNBC is a rational target for immunotherapy due to high PD-L1 expression on tumor-infiltrating immune cells (IC) and elevated T cell tumor infiltration. Furthermore, combining chemotherapy with ICI is hypothesized to enhance anti-tumor immune response via neoantigen release.
A randomized, placebo-controlled study will be included in each group.
Participant characteristics: locally advanced or metastatic TNBC, ≥18 years.

30. * Type and method of review.

Select the type of review, review method and health area from the lists below.

Type of review

Cost effectiveness	No
Diagnostic	No

Epidemiologic	No
Individual patient data (IPD) meta-analysis	No
Intervention	No
Living systematic review	No
Meta-analysis	Yes
Methodology	No
Narrative synthesis	No
Network meta-analysis	No
Pre-clinical	No
Prevention	No
Prognostic	No
Prospective meta-analysis (PMA)	No
Review of reviews	No
Service delivery	No
Synthesis of qualitative studies	No
Systematic review	Yes
Other	No

Health area of the review

Alcohol/substance misuse/abuse	No
Blood and immune system	No
Cancer	Yes
Cardiovascular	No
Care of the elderly	No
Child health	No
Complementary therapies	No
COVID-19	No
Crime and justice	No
Dental	No
Digestive system	No
Ear, nose and throat	No

Education	No
Endocrine and metabolic disorders	No
Eye disorders	No
General interest	No
Genetics	No
Health inequalities/health equity	No
Infections and infestations	No
International development	No
Mental health and behavioural conditions	No
Musculoskeletal	No
Neurological	No
Nursing	No
Obstetrics and gynaecology	No
Oral health	No
Palliative care	No
Perioperative care	No
Physiotherapy	No
Pregnancy and childbirth	No
Public health (including social determinants of health)	No
Rehabilitation	No
Respiratory disorders	No
Service delivery	No
Skin disorders	No
Social care	No
Surgery	No
Tropical Medicine	No
Urological	No
Wounds, injuries and accidents	No
Violence and abuse	No

31. Language.

Select each language individually to add it to the list below, use the bin icon to remove any added in error.

English

There is not an English language summary

32. * Country.

Select the country in which the review is being carried out. For multi-national collaborations select all the countries involved.

China

33. Other registration details.

Name any other organisation where the systematic review title or protocol is registered (e.g. Campbell, or The Joanna Briggs Institute) together with any unique identification number assigned by them.
If extracted data will be stored and made available through a repository such as the Systematic Review Data Repository (SRDR), details and a link should be included here. If none, leave blank.

34. Reference and/or URL for published protocol. [1 change]

If the protocol for this review is published provide details (authors, title and journal details, preferably in Vancouver format)

No I do not make this file publicly available until the review is complete

35. Dissemination plans.

Do you intend to publish the review on completion?

No

36. Keywords.

Give words or phrases that best describe the review. Separate keywords with a semicolon or new line. Keywords help PROSPERO users find your review (keywords do not appear in the public record but are included in searches). Be as specific and precise as possible. Avoid acronyms and abbreviations unless these are in wide use.

Immune checkpoint inhibitor
Adjuvant chemotherapy
Triple-negative breast cancer
Pembrolizumab
Atezolizumab
Durvalumab

37. Details of any existing review of the same topic by the same authors.

If you are registering an update of an existing review give details of the earlier versions and include a full bibliographic reference, if available.

38. * Current review status.

Update review status when the review is completed and when it is published.
New registrations must be ongoing so this field is not editable for initial submission.

Review_Ongoing

39. Any additional information.

Provide any other information relevant to the registration of this review.

40. Details of final report/publication(s) or preprints if available.

Leave empty until publication details are available OR you have a link to a preprint (NOTE: this field is not editable for initial submission).
List authors, title and journal details preferably in Vancouver format.
