# Supplementary material for: Addition of PD-1/PD-L1 inhibitors to chemotherapy for triple-negative breast cancer: a meta-analysis
Source: Front Oncol. 2024 Feb 9;14:1309677. doi: 10.3389/fonc.2024.1309677 (PMC10884307; doi:10.3389/fonc.2024.1309677)
Supplement: Supplementary file 2 [file DataSheet_2.pdf]

## Supplementary Data 2. Search strategy

## PubMed search strategy

#1 "Triple Negative Breast Neoplasms"[Mesh]

#2 ((((Triple Negative Breast cancer*[Title/Abstract]) OR (Triple Negative Breast neoplasm*[Title/Abstract])) OR (Triple Negative Breast carcinoma*[Title/Abstract ])) OR (Triple Negative Breast tumo?r*[Title/Abstract])) OR (TNBC[Title/Abstract])

#3 #1 OR #2

#4 ((((programmed cell death 1 receptor[MeSH Terms]) OR (programmed cell death 1 ligand 2 protein[MeSH Terms])) OR (immunotherapy[MeSH Terms])) OR (Immune Checkpoint Inhibitors[MeSH Terms])) OR (Ipilimumab[MeSH Terms])

#5 ((((((((((((((((((((((((((((((((((((((((((((Programmed Cell Death 1[Title/Abstract]) OR (PD-1 Receptor[Title/Abstract])) OR (CD279 Antigen*[Title/Abstract])) OR (PD1 Receptor[Title/Abstract])) OR (CD273 Antigen[Title/Abstract])) OR (PD L2 Ligand[Title/Abstract])) OR (B7 DC Ligand[Title/Abstract])) OR (B7 DC Antigen*[Title/Abstract])) OR (programmed death[Title/Abstract])) OR (pd l1[Title/Abstract])) OR (pd l2[Title/Abstract])) OR (pd 2[Title/Abstract])) OR (immunotherap*[Title/Abstract])) OR (durvalumab[Title/Abstract])) OR (MEDI4736[Title/Abstract])) OR (MEDI -4736[Title/Abstract])) OR (Imfinzi[Title/Abstract])) OR (avelumab[Title/Abstract])) OR (MSB0010718C[Title/Abstract])) OR (atezolizumab[Title/Abstract])) OR (MPDL3280A[Title/Abstract])) OR (Tecentriq[Title/Abstract])) OR (RG7446[Title/Abstract])) OR (pembrolizumab[Title/Abstract])) OR (lambrolizumab[Title/Abstract])) OR (Keytruda[Title/Abstract])) OR (cemiplimab[Title/Abstract])) OR (MK3475[Title/Abstract])) OR (MK 3475[Title/Abstract])) OR (REGN2810[Title/Abstract])) OR (nivolumab[Title/Abstract])) OR (MDX-1106[Title/Abstract])) OR (ONO-4538[Title/Abstract])) OR (BMS-936558[Title/Abstract])) OR (Opdivo[Title/Abstract])) OR (Immune Checkpoint Inhibit*[Title/Abstract])) OR (Immune Checkpoint Block*[Title/Abstract])) OR (ICI[Title/Abstract])) OR (Yervoy[Title/Abstract])) OR (MDX 010[Title/Abstract])) OR (MDX010[Title/Abstract])) OR (MDX CTLA 4[Title/Abstract])) OR (tremelimumab[Title/Abstract])) OR (ticilimumab[Title/Abstract])) OR (CP 675*[Title/Abstract])

#6 #4 OR #5

#7 (randomized controlled trial[pt] OR controlled clinical trial[pt] OR randomized[tiab] OR placebo[tiab] OR clinical trials as topic[mesh:noexp] OR randomly[tiab] OR trial[ti]) NOT (animals [mh] NOT (humans [mh] AND animals[mh])) 1299978

#8 #3 AND #6 AND #7

## EMBASE search strategy

#1 'triple negative breast cancer'/exp

#2 'triple negative breast cancer*':ab,ti OR 'triple negative breast neoplasm*':ab,ti OR 'triple negative breast carcinoma*':ab,ti OR 'triple negative breast tumo?r*':ab,ti OR tnbc:ab,ti

#3 #1 OR #2

#4 'programmed death 1 receptor'/exp

#5 'programmed death 1 ligand 2'/exp

#6 'immunotherapy'/exp

#7 'immune checkpoint inhibitor'/exp

#8 'a prospective parallel design study testing non-inferiority of customized oral stents made using 3d printing':sp,ab,ti OR 'manually fabricated methods':sp,ab,ti OR 'a prospective parallel design study testing non-inferiority of customized oral stents made using 3d printing':ti,ab OR 'manually fabricated methods':ti,ab OR 'programmed cell death 1':ab,ti OR 'pd -1 receptor':ab,ti OR 'cd279 antigen*':ab,ti OR 'pd1 receptor':ab,ti OR 'cd273 antigen':ab,ti OR 'pd l2 ligand':ab,ti OR 'b7 dc ligand':ab,ti OR 'b7 dc antigen*':ab,ti OR 'programmed death':ab,ti OR 'pd l1':ab,ti OR 'pd l2':ab,ti OR 'pd 2':ab,ti OR immunotherap*:ab,ti OR durvalumab:ab,ti OR medi4736:ab,ti OR 'medi 4736':ab,ti OR imfinzi:ab,ti OR avelumab:ab,ti OR msb0010718c:ab,ti OR atezolizumab:ab,ti OR mpdl3280a:ab,ti OR tecentriq:ab,ti OR rg7446:ab,ti OR pembrolizumab:ab,ti OR lambrolizumab:ab,ti OR keytruda:ab,ti OR cemiplimab:ab,ti OR mk3475:ab,ti OR 'mk 3475':ab,ti OR regn2810:ab,ti OR nivolumab:ab,ti OR 'mdx 1106':ab,ti OR 'ono 4538':ab,ti OR 'bms 936558':ab,ti OR opdivo:ab,ti OR 'immune checkpoint inhibit*':ab,ti OR 'immune checkpoint block*':ab,ti OR ici:ab,ti OR yervoy:ab,ti OR 'mdx 010':ab,ti OR mdx010:ab,ti OR 'mdx ctla 4':ab,ti OR ticilimumab:ab,ti OR 'cp 675*':ab,ti OR cp675*:ab,ti OR tremelimumab:ab,ti

#9 #4 OR #5 OR #6 OR #7 OR #8

#10 'crossover procedure':de OR 'double-blind procedure':de OR 'randomized controlled trial':de OR 'single-blind procedure':de OR random*:de,ab,ti OR factorial*:de,ab,ti OR crossover*:de,ab,ti OR ((cross NEXT/1 over*):de,ab,ti) OR placebo*:de,ab,ti OR ((doubl* NEAR/1 blind*):de,ab,ti) OR ((singl* NEAR/1 blind*):de,ab,ti) OR assign*:de,ab,ti OR allocat*:de,ab,ti OR volunteer*:de,ab,ti

#11 #3 AND #9 AND #10

#12 #11 AND [embase]/lim NOT ([embase]/lim AND [medline]/lim)

## Cochrane Central Register of Controlled Trials search strategy

#1 MeSH descriptor: [Triple Negative Breast Neoplasms] explode all trees

#2 (Triple Negative Breast cancer* OR Triple Negative Breast neoplasm* OR Triple Negative Breast carcinoma* OR Triple Negative Breast tumo?r* OR TNBC):ti,ab,kw

#3 #1 OR #2

#4 MeSH descriptor: [Programmed Cell Death 1 Receptor] explode all trees

#5 MeSH descriptor: [Programmed Cell Death 1 Ligand 2 Protein] explode all trees

#6 MeSH descriptor: [Immunotherapy] explode all trees

#7 MeSH descriptor: [Immune Checkpoint Inhibitors] explode all trees

#8 MeSH descriptor: [Ipilimumab] explode all trees

#9 (Programmed Cell Death 1 OR PD-1 Receptor OR CD279 Antigen* OR PD1 Receptor OR CD273 Antigen OR PD L2 Ligand OR B7 DC Ligand OR B7 DC Antigen* OR programmed death OR pd l1 OR pd l2 OR pd 2 OR immunotherap* OR durvalumab OR MEDI4736 OR MEDI -4736 OR Imfinzi OR avelumab OR MSB0010718C OR atezolizumab OR MPDL3280A OR Tecentriq OR RG7446 OR pembrolizumab OR lambrolizumab OR Keytruda OR cemiplimab OR MK3475 OR MK 3475 OR REGN2810 OR nivolumab OR MDX -1106 OR ONO-4538 OR BMS-936558 OR Opdivo OR Immune Checkpoint Inhibit* OR Immune Checkpoint Block* OR ICI OR Yervoy OR MDX 010 OR MDX010 OR MDX CTLA 4 OR tremelimumab OR ticilimumab OR CP 675* OR CP675*):ti,ab,kw

#10 #4 OR #5 OR #6 OR #7 OR #8 OR #9

#11 #3 AND #10

## Web of Science search strategy

TS=(Triple Negative Breast Neoplasms or Triple Negative Breast cancer* or Triple Negative Breast neoplasm* or Triple Negative Breast carcinoma* or oftriple Negative Breast tumo?r* or TNBC) | 28，481

Programmed Cell Death 1 OR PD-1 Receptor OR CD279 Antigen* OR PD1 Receptor OR CD273 Antigen OR PD L2 Ligand OR B7 DC Ligand OR B7 DC Antigen* OR programmed death OR pd l1 OR pd l2 OR pd 2 OR immunotherap* OR durvalumab OR MEDI4736 OR MEDI -4736 OR Imfinzi OR avelumab OR MSB0010718C OR atezolizumab OR MPDL3280A OR Tecentriq OR RG7446 OR pembrolizumab OR lambrolizumab OR Keytruda OR cemiplimab OR MK3475 OR MK 3475 OR REGN2810 OR nivolumab OR MDX-1106 OR ONO- 4538 OR BMS-936558 OR Opdivo OR Immune Checkpoint Inhibit*OR Immune Checkpoint Block*OR ICI OR Yervoy OR MDX 010 OR MDX010 OR MDX CTLA 4 OR tremelimumab OR ticilimumab OR CP 675*
