# Supplementary figures and images for: Addition of PD-1/PD-L1 inhibitors to chemotherapy for triple-negative breast cancer: a meta-analysis
Source: Front Oncol. 2024 Feb 9;14:1309677. doi: 10.3389/fonc.2024.1309677 (PMC10884307; doi:10.3389/fonc.2024.1309677)

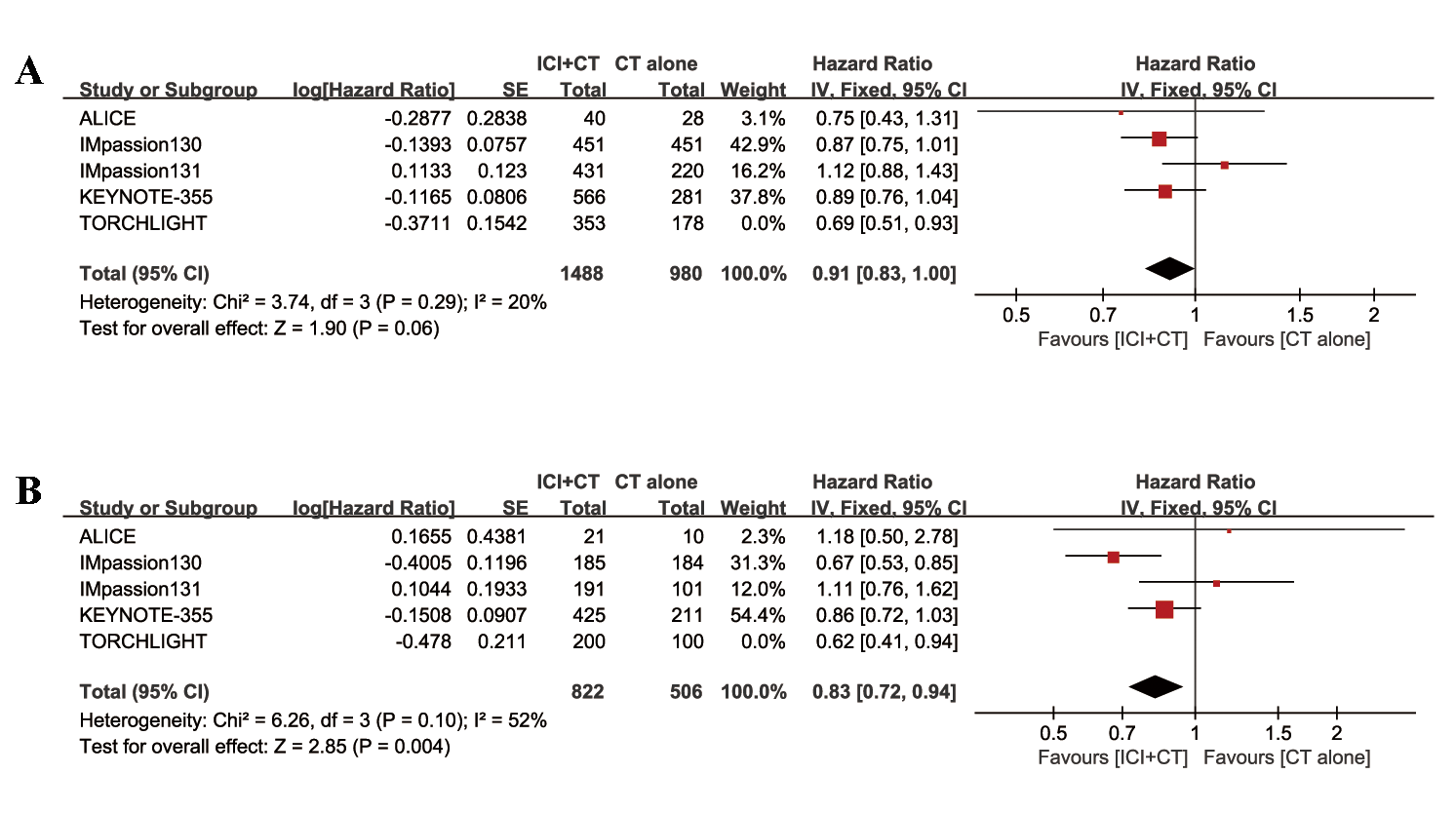

Supplement: Supplementary file 3 [file Image_1.tif]

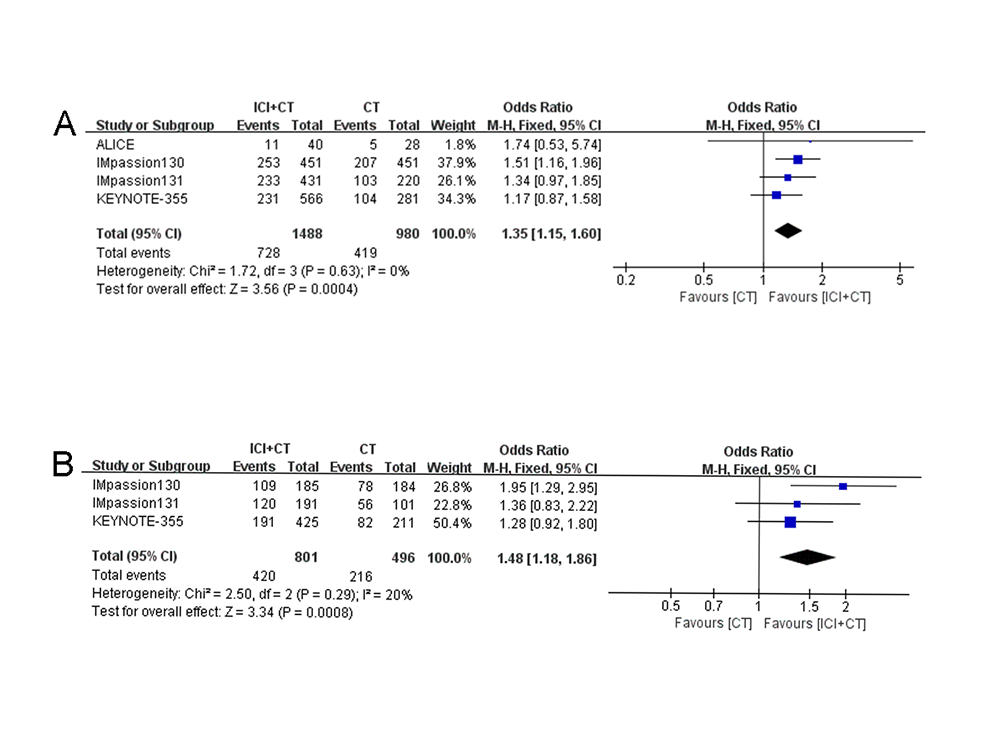

Supplement: Supplementary file 4 [file Image_2.tif]
